# The immunogenicity of recombinant vaccines based on modified Vaccinia Ankara (MVA) viruses expressing African horse sickness virus VP2 antigens depends on the levels of expressed VP2 protein delivered to the host

**DOI:** 10.1016/j.antiviral.2018.04.015

**Published:** 2018-06

**Authors:** Eva Calvo-Pinilla, Simon Gubbins, Peter Mertens, Javier Ortego, Javier Castillo-Olivares

**Affiliations:** a The Pirbright Institute, Ash Road, Pirbright, Surrey, GU24 0NF, UK; b INIA-CISA, 28130, Valdeolmos, Madrid, Spain

**Keywords:** African horse sickness, Vaccinia, MVA, VP2

## Abstract

African horse sickness (AHS) is a lethal equine disease transmitted by *Culicoides* biting midges and caused by African horse sickness virus (AHSV). AHS is endemic to sub-Saharan Africa, but devastating outbreaks have been recorded periodically outside this region. The perceived risk of an AHS outbreak occurring in Europe has increased following the frequent epidemics caused in ruminants by bluetongue virus, closely related to AHSV.

Attenuated vaccines for AHS are considered unsuitable for use in non-endemic countries due bio-safety concerns. Further, attenuated and inactivated vaccines are not compatible with DIVA (differentiate infected from vaccinated animals) strategies. All these factors stimulated the development of novel AHS vaccines that are safer, more efficacious and DIVA compatible.

We showed previously that recombinant modified Vaccinia Ankara virus (MVA) vaccines encoding the outer capsid protein of AHSV (AHSV-VP2) induced virus neutralising antibodies (VNAb) and protection against AHSV in a mouse model and also in the horse. Passive immunisation studies demonstrated that immunity induced by MVA-VP2 was associated with pre-challenge VNAb titres in the vaccinates. Analyses of the inoculum of these MVA-VP2 experimental vaccines showed that they contained pre-formed AHSV-VP2.

We continued studying the influence of pre-formed AHSV-VP2, present in the inoculum of MVA-VP2 vaccines, in the immunogenicity of MVA-VP2 vaccines. Thus, we compared correlates of immunity in challenged mice that were previously vaccinated with: a) MVA-VP2 (live); b) MVA-VP2 (live and sucrose gradient purified); c) MVA-VP2 (UV light inactivated); d) MVA-VP2 (UV light inactivated and diluted); e) MVA-VP2 (heat inactivated); f) MVA-VP2 (UV inactivated) + MVA-VP2 (purified); g) MVA-VP2 (heat inactivated) + MVA-VP2 (purified); and h) wild type-MVA (no insert). The results of these experiments showed that protection was maximal using MVA-VP2 (live) vaccine and that the protection conferred by all other vaccines correlated strongly with the levels of pre-formed AHSV-VP2 in the vaccine inoculum.

## Highlights

• MVA-VP2 vaccines (expressing African horse sickness virus VP2) induce protective immunity in mouse model and in horses.
• Experimental MVA-VP2 vaccines contain preformed AHSV-VP2 in the inoculum if they are not sucrose-gradient purified.
• MVA-VP2 vaccines express AHSV-VP2 in MVA-VP2 infected cells.
• Both pre-formed AHSV-VP2 and ‘*de novo*’ synthesised AHSV-VP2 in MVA-VP2 vaccinates contribute to MVA-VP2 immunogenicity.

## 1 Introduction

African horse sickness (AHS) is an arthropod-borne viral disease of solipeds transmitted by haematophagus insects of the genus *Culicoides*, the horse being the most severely affected species. The disease is caused by African horse sickness virus (AHSV), a member of the genus *Orbivirus*, family *Reoviridae*, closely related to bluetongue virus (BTV). The non-enveloped 55–70 nm AHSV spherical virion consists of a triple-layered capsid surrounding ten double-stranded RNA segments and three proteins involved in viral replication (VP1, VP4 and VP6, encoded by segments 1, 4 and 9 respectively) (Roy et al., 1994, Manole et al., 2012). The inner capsid protein VP3, encoded by segment 3, forms the icosahedral scaffold to which trimers of the conserved VP7 protein bind. The outer capsid is formed by two major structural proteins, VP2 and VP5 (encoded by segments 2 and 6 respectively), involved in cell attachment and entry. VP2 is the most variable antigen of AHSV, determines serotype formation (Burrage et al., 1993) and contains most of the virus neutralising antibody (VNAb) epitopes identified so far (Bentley et al., 2000, Burrage et al., 1993, Martinez-Torrecuadrada et al., 1999).

To date no effective treatment exists for AHS and, consequently, control of the disease relies on vaccination, control of animal movements and prevention of bites by *Culicoides* midges. Live attenuated AHS vaccines (LAV) have been in use in Africa for almost 100 years and permitted the subsistence of horses in that part of the world (Coetzer and Guthrie, 2004; Mellor and Hamblin, 2004, von Teichman and Smit, 2008). There are nine different serotypes of AHS virus (AHSV) and protective immunity is long-lived but serotype-specific. Despite their apparent efficacy, the use of LAV presents a series of bio-safety concerns, especially in non-endemic countries, which arise from the ability of vaccine viruses to replicate *in vivo* and to exchange their genome segments with other vaccine or field AHS viruses. Recent studies indicate that outbreaks of AHS occurring in the Western Cape Province of South Africa between 2004 and 2014 may have resulted from re-assortment events involving vaccine strains of serotype 1 (Weyer et al., 2016).

Over the last 30 years much attention has been given to the development of safer alternative AHS vaccines and a number of different approaches have been explored. These included the use of inactivated AHSV (House et al., 1994, Lelli et al., 2013), baculovirus-expression of AHSV capsid proteins (Roy, 1996), plasmid DNA vaccines (Romito et al., 1999) or poxvirus expression vectors (Alberca et al., 2014, Castillo-Olivares et al., 2011, de la Poza et al., 2013, Guthrie et al., 2009). In previous studies we showed that recombinant modified Vaccinia Ankara (MVA) viruses expressing AHSV-VP2 induced VNAb and complete clinical protection in a mouse model and in the equine species (Alberca et al., 2014, Castillo-Olivares et al., 2011, de la Poza et al., 2013).

There is evidence suggesting that cell-mediated immune responses play an important role in AHS immunity. Cell-mediated immune responses have been detected in horses immunized with live attenuated vaccines (Pretorius et al., 2012) or recombinant Canarypox viruses expressing VP2 and VP5 (El Garch et al., 2012) and, more recently, in interferon alpha receptor gene knock-out mice (IFNAR −/−) after vaccination with MVA VP2/NS1 (de la Poza et al., 2013). However, we demonstrated that the effector mechanisms of immunity of MVA-VP2 vaccination in mice are mediated mainly by antibodies (Calvo-Pinilla et al., 2014, Calvo-Pinilla et al., 2015).

Recombinant MVA vaccine viruses are replication-deficient in mammalian cells and clearance of MVA and its genes occurs rapidly following inoculation (Altenburg et al., 2014). Despite the transient expression of MVA encoded proteins within the vaccinated host, recombinant MVA vaccines efficiently induce cellular and humoral immunity (Drexler et al., 2004, Sutter et al., 1994). Thus, induction of AHSV-VP2-specific antibody responses after MVA-VP2 vaccination is thought to depend on expression of VP2 protein from MVA-VP2 infected cells.

In our previous studies, MVA-VP2 vaccines were administered as MVA-VP2-infected DF-1 cell lysates. Thus, pre-formed VP2 was believed to be present in the inoculum of these vaccines. In this paper, we describe the results of experiments aimed at determining the role that both pre-formed VP2, and VP2 synthesised *de novo* from MVA-VP2 infected host cells, play in the immunogenicity of MVA-VP2 vaccines.

## 2 Materials and methods

### 2.1 Viruses and cells

Vero cells (ATCC, Cat. No. CCL-81) and Chicken embryo fibroblast (DF-1) (ATCC, Cat. No. CRL-12203) were grown in high glucose Dulbecco's modified Eagle's medium (DMEM) supplemented with 2 mM glutamine, penicillin (100 units/ml), streptomycin (100 μg/ml) and 10% foetal calf serum (FCS). AHSV serotype 4 (AHSV-4, Madrid/87) was grown in Vero cells and MVA viruses (wild type-MVA and MVA-VP2) grown in DF-1 cells. Virus stocks were generated by infection of sub confluent cells using a multiplicity of infection (MOI) of 0.1. When a total cytopathic effect (CPE) was visible, the cells and supernatants were harvested and centrifuged. The virus was released from the cells by three freeze/thaw cycles, sonication and then titrated by plaque assay.

### 2.2 MVA-VP2 vaccine preparations

Various MVA-VP2 vaccine preparations were used:

a) MVA-VP2 (live): MVA-VP2, expressing AHSV-4 VP2, was previously described (Castillo-Olivares et al., 2011, Chiam et al., 2009) and was bulked up for this study in DF-1 cells and subsequently used as a cell lysate.
b) MVA-VP2 (UV) was obtained by exposure of 1 ml aliquots of MVA-VP2 dispensed on a p100 tissue culture plate to 254 nm wavelength UV light for 40 min. UV irradiation was achieved by placing the plates at 3 cm below a TUV T8 lamp (Philips).
c) MVA-VP2 (HI), was heat-inactivated by heating 100 μl MVA-VP2 aliquots in a thermal block for 15 min at 56 °C.
d) MVA-VP2 (SGP) is a sucrose-gradient-purified MVA-VP2 preparation obtained as follows. A volume of 20 ml of MVA-VP2 was overlaid carefully on top of 20 ml of a sterile 36% (w/w) solution of sucrose in PBS using 40 ml ultra-clear centrifuge tube (Beckmann). The samples were centrifuged for 1.5 h at 300000 g and 4 °C in a SW 28 rotor. The pellets were re-suspended thoroughly in 0.5 ml sterile PBS and sonicated briefly before titration.

### 2.3 MVA-VP2 inactivation test

MVA-VP2 (HI) and MVA-VP2 (UV) were tested for infectivity using MVA-VP2 (live) as a positive control. All three preparations were inoculated (MOI of 1) into DF-1 cell cultures. After an adsorption period of 1.5 h cells were washed and medium was replaced. After 48 h, infected cells were washed three times with PBS and RNA was extracted from the cells using Trizol reagent (Invitrogen) according to the method recommended by the manufacturer.

Reverse transcription-PCR (RT-PCR) was used to detect mRNA from AHSV-4 genome segment 2 (coding for VP2 protein) in DF-1 cells incubated with live and inactivated MVA-VP2. Oligonucleotide primers used to amplify a segment of 900 bp were: a) forward primer: 5′-CGCCCGGGATGGCGTCCGAGTTTGGAATATTG-3´; and b) reverse primer: 5′-CGCCCGGGCTACCCCTGCTTATCACCTGCTGA-3´.

RNA was denatured in the presence of a reverse VP2 gene-specific primer and dNTP mix by heating to 65 °C for 5 min. A mix of RT buffer, MgCl_2_ and Reverse Transcriptase (200 U/μL) was added and the reaction was incubated for 1 h at 50 °C. Amplification of the VP2 gene was performed by PCR using PCR Buffer II (Invitrogen), dNTPs, specific primers (forward and reverse primers), MgCl_2_ solution, AmpliTaq DNA Polymerase (Invitrogen), and cDNA template. Amplification cycle parameters were: 94 °C for 2 min (1×); 94 °C for 45 s, 55 °C for 1 min, and 72 °C for 2 min (30×); 94 °C for 15 min (1×).

### 2.4 Western blot

Immunoblotting was performed as described previously (Chiam et al., 2009). Samples were mixed 1:1 with 2× Laemmli sample buffer and 12 μL were loaded in each well of polyacrylamide gels. Three immunogenic AHSV-4 VP2-derived KLH-conjugated peptides (NH_2_-KKKEEGEDDTARQEIRKAWC-COOH; NH_2_-NKGKWKEHIKEVTEKLKKA-COOH; NH_2_-DMNEKQKPYFEFEYDDFKPC-COOH) were selected to obtain a VP2-specific rabbit polyclonal antibody from a commercial source (GenScript). This antibody was used at a 1:400 dilution. A goat anti-rabbit peroxidase (Bio-Rad) was used at a dilution of 1:10000.

### 2.5 Mice

Seven-week-old, female, Type I interferon receptor KO A129 IFNAR (−/−) mice were purchased from B&K Universal. The animals were rested for about a week before the experiments were performed in the animal facilities of the Centro de Investigación en Sanidad Animal (INIA-CISA). All protocols for animal use were approved by the Ethical Committee of the Centre for Animal Health Research (CISA-INIA) (Permit number: PROEX 039/15) in strict accordance with the Spanish National Royal Decree (RD1201/2005), EU guidelines 2010/63/UE about protection of animals used for experimentation and other scientific purposes, and the Spanish Animal Welfare Act 32/2007.

### 2.6 Vaccination of animals

Mice (n = 5) were inoculated twice with 10^7^ pfu/mouse (or equivalent in the case of inactivated vaccines) of each vaccine preparation for the first experiment. For the second experiment, 3 groups of mice (n = 5) were vaccinated with 10^7^, 10^8^ or 5 × 10^8^ pfu/mouse of purified MVA-VP2. All immunisations were performed at day 0 and 21 by the intra-peritoneal route. Sera were collected from the submandibular vein at 2 weeks after the second vaccination for serological analysis.

### 2.7 Plaque reduction neutralization test (PRNT)

Serial dilutions of mouse sera were incubated with 100 TCID_50_ of AHSV-4 for 1 h at 37 °C. Then, samples were inoculated into one well each of 12-well plates containing confluent monolayers of Vero cells. Following incubation for 1 h at 37 °C in 5% CO_2_ an agar overlay (DMEM, 10% FBS, 0.4% agar) was added and plates incubated further for 4 days at 37 °C in 5% CO_2_. Plaques were visualized with a counter-stain solution (2% crystal violet, 10% formaldehyde, PBS). PRNT titre was calculated as the reciprocal (log_10_) of the highest dilution of serum that neutralised 50% of the control virus input.

### 2.8 Challenge with AHSV

Two weeks after the second vaccination, mice from the first experiment were infected with 10^6^ TCID_50_ of AHSV-4 per mouse administered by the sub-cutaneous route. Animals were monitored twice a day after challenge.

### 2.9 Clinical signs and viraemia

Clinical scoring was performed as described previously (Calvo-Pinilla et al., 2015). Mice were humanely euthanized when they showed severe clinical signs (weight loss, dehydration, frequent hunching, severe conjunctivitis or any other condition that prevented food or water intake).

Whole-EDTA-blood samples were collected from the submandibular vein at different days post infection to analyse viraemia. Blood cells were lysed with water before performing plaque assays on Vero cells as described previously (Castillo-Olivares et al., 2011).

### 2.10 Statistical analysis

In the first experiment, differences in survival amongst groups of mice receiving each vaccine were compared using log-rank tests. Clinical scores in groups of mice receiving each vaccine were compared using Kruskal-Wallis tests. Differences between groups were then explored using post hoc Wilcoxon rank-sum tests. Differences in viraemia amongst groups of mice receiving each vaccine were assessed using linear mixed models with log10 pfu + 1 as the response variable, days post infection (as a factor) and group as fixed effects and mouse as a random effect. Model selection proceeded by stepwise deletion of non-significant terms (as judged by the Akaike information criterion) starting from a model including days post infection and group as main effects. In the second experiment, the relationship between vaccine dose and PRNT_50_ titre was explored using linear regression with log_10_ PRNT_50_ titre as the response variable and log_10_ dose as the explanatory variable. A significance level of P = 0.05 was used in all analyses. All methods were implemented in R version 3.2.1 (R Core Team 2015).

## 3 Results

In order to investigate the immunogenicity of MVA-VP2 vaccines in relation to pre-formed AHSV-VP2 present in the inoculum, we conducted two vaccination experiments using various MVA-VP2 vaccine preparations. In the first vaccination experiment we vaccinated 8 groups of 5 mice with either of the following: a) MVA-VP2 (live); b) MVA-VP2 (SGP); c) MVA-VP2 (UV); d) MVA-VP2 (UV 1/4) prepared by diluting MVA-VP2 (UV) in DMEM; e) MVA-VP2 (HI); f) MVA-VP2 (UV) + MVA-VP2 (SGP); g) MVA-VP2 (HI) + MVA-VP2 (SGP); and h) wild type-MVA (no insert). Following vaccination, animals were challenged with AHSV-4 and protective immunity assessed. In the second experiment, we vaccinated 3 groups of 5 mice with either 10^7^ pfu/mouse; 10^8^ pfu/mouse or 5 × 10^8^ pfu/mouse of MVA-VP2 (SGP). Sera were obtained from these animals after vaccination and VNAb titres determined.

Some of these MVA-VP2 vaccines were generated by inactivating the infectivity of MVA-VP2 cell lysate stocks by exposure to heat or UV light. Therefore, the immunogenicity of these vaccines would rely only on the presence of pre-formed AHSV-VP2 in the inoculum and not on VP2 protein synthesised *de novo* from MVA-VP2 infected host cells.

### 3.1 Inactivation of MVA-VP2 (HI) and MVA-VP2 (UV)

**Fig. 1:**
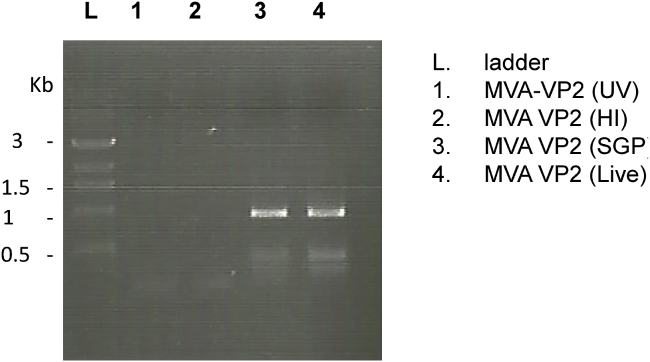
**Inactivation of MVA-VP2 by heat or UV irradiation**. Agarose gel electrophoresis of RT-PCR product of the AHSV-VP2 gene transcripts. DF-1 cells were infected with live, purified, heat or UV irradiated MVA-VP2 and 48 h later, VP2-mRNA detection was performed by RT-PCR.

Both inactivated MVA-VP2 vaccines did not produce cytopathic effect in DF-1 cell cultures after an incubation period of 5 days. In order to corroborate that inactivation of infectivity was successful and that live MVA-VP2 virus was not present in the inoculum of MVA-VP2 (HI) and MVA-VP2 (UV) vaccines, fresh DF-1 cell cultures were inoculated with MVA-VP2 (HI) or MVA-VP2 (UV) and 48 h later VP2-mRNA detection was performed by RT-PCR. PCR products of the expected size could only be obtained from MVA-VP2 (SGP) and positive control MVA-VP2 (live) infected cultures (Fig. 1). Additional experiments (not shown) indicated that total MVA-VP2 inactivation was achieved after UV (λ 254 nm) exposure for 40 min or by heat treatment at 56 °C for 15 min.

### 3.2 Antigenicity of AHSV-VP2 present in the inoculum

**Fig. 2:**
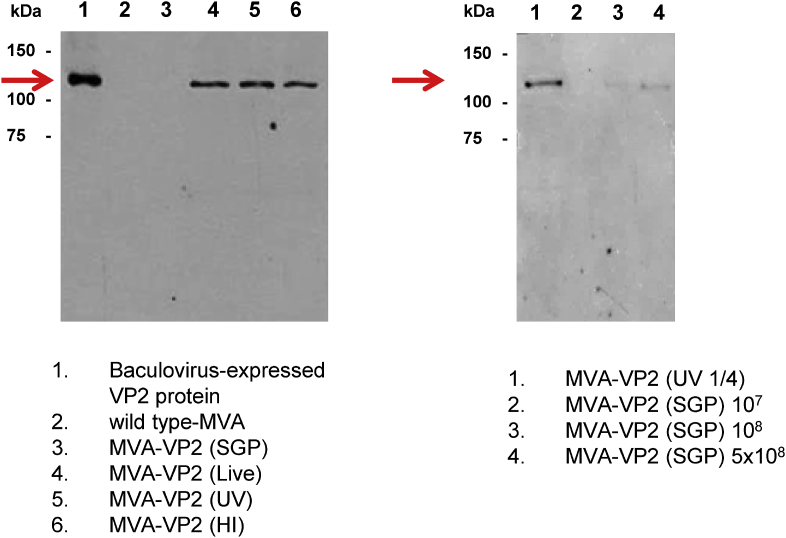
**Presence of AHSV-VP2 protein in the MVA-VP2 vaccines’ preparations**. Immunoblotting of vaccine inoculum (left panel: first vaccination experiment; right panel: second vaccination experiment) was performed using a VP2-specific rabbit polyclonal antibody following SDS-PAGE separation of proteins. Arrowhead indicates the expected size of AHSV-4 VP2.

All the vaccine preparations used in the first mouse experiment were analysed for the presence of VP2 in the inoculum by immuno-blotting with VP2-specific rabbit polyclonal antisera. The results indicated that, with the exception of MVA-VP2 (SGP) and MVA (wt), all samples showed a clear band of approximately 116 kDa, which corresponds to monomers of AHSV-VP2 (Fig. 2).

### 3.3 Vaccination and challenge experiment with live and inactivated MVA-VP2 vaccines

Protective immunity against AHSV-4 conferred by each of the MVA-VP2 vaccine preparations was assessed in a vaccination and challenge experiment.

#### 3.3.1 Antibody responses following vaccination

**Fig. 3:**
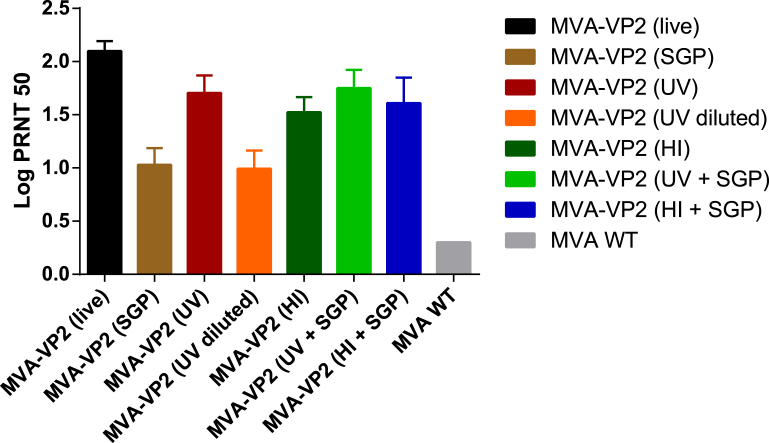
**Virus neutralizing antibody (VNAb) responses to AHSV in vaccinated mice**. Serum VNAb were determined by PRNT. Titers were assigned arithmetically as the dilution of serum that gave a 50% neutralization endpoint and expressed as log_10_ values. Each bar represents the mean of antibody titers of individual serum samples collected on day 35 post-immunization. Error bars represent the standard deviation within the samples.

Determination of VNAb titres against AHSV-4 of post-vaccination (day 35) sera showed that all MVA-VP2 vaccinates developed VNAb. However, there were significant differences between some groups (Fig. 3). Highest values were recorded for MVA-VP2 (live) vaccinates (PRNT_50_ = 2.10), followed by those obtained from all groups that were vaccinated with preparations containing un-diluted inactivated MVA-VP2 (PRNT_50_ titres ranging between 1.52 and 1.75). As expected, sera from MVA-VP2 (1/4 UV) vaccinates showed PRNT_50_ titres just below the positive cut-off value of the assay (PRNT_50_ = 0.99). Similar values were recorded for MVA-VP2 (SGP) vaccinates (PRNT_50_ = 1.03). The data collectively suggest that the presence of pre-formed AHSV-VP2 in the inactivated MVA-VP2 vaccines played an important role in their capacity to induce a VNAb response and that the immunogenicity was reduced when the pre-formed VP2 in the inoculum was diluted or removed from the inoculum of live MVA-VP2 vaccines (at the dose of 10^7^ pfu/mouse).

#### 3.3.2 Protection against AHSV-4 challenge

All vaccinates exhibited survival rates of 100% (five out of five mice surviving), except those receiving MVA-VP2 (SGP) and MVA-VP2 (¼ UV) (both 80%, four out of five mice surviving). As expected, all MVA (wt) vaccinated mice succumbed to AHSV-4 challenge. Survival was significantly (P < 0.001) lower in mice receiving wild-type MVA compared with mice receiving any of the other vaccines.

**Fig. 4:**
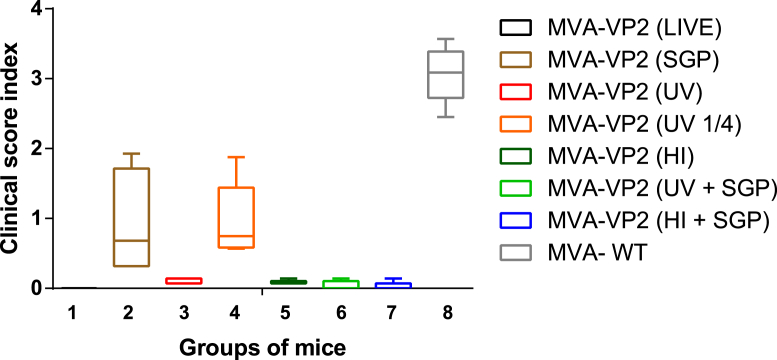
**Clinical score index after challenge**. The clinical signs were recorded daily for 14 days. The lines within the box represents the mean of clinical score index within the group. Error bars represent the standard deviation.

Clinical scores (Fig. 4) differed significantly amongst groups (P < 0.001), they are in line with survival rates and can be ranked in decreasing order as follows: MVA (wt) ^a^ > MVA-VP2 (1/4 UV) ^b^ > MVA-VP2 (SGP) ^b^ > MVA-VP2 (HI) ^c^ > MVA-VP2 (UV) ^c^ > MVA-VP2 (SGP) + MVA-VP2 (UV) ^cd^ > MVA-VP2 (SGP) + MVA-VP2 (HI) ^d^ > MVA-VP2 (live) ^d^ (where groups share a common superscripted letter they do not differ significantly (P > 0.05)).

**Fig. 5:**
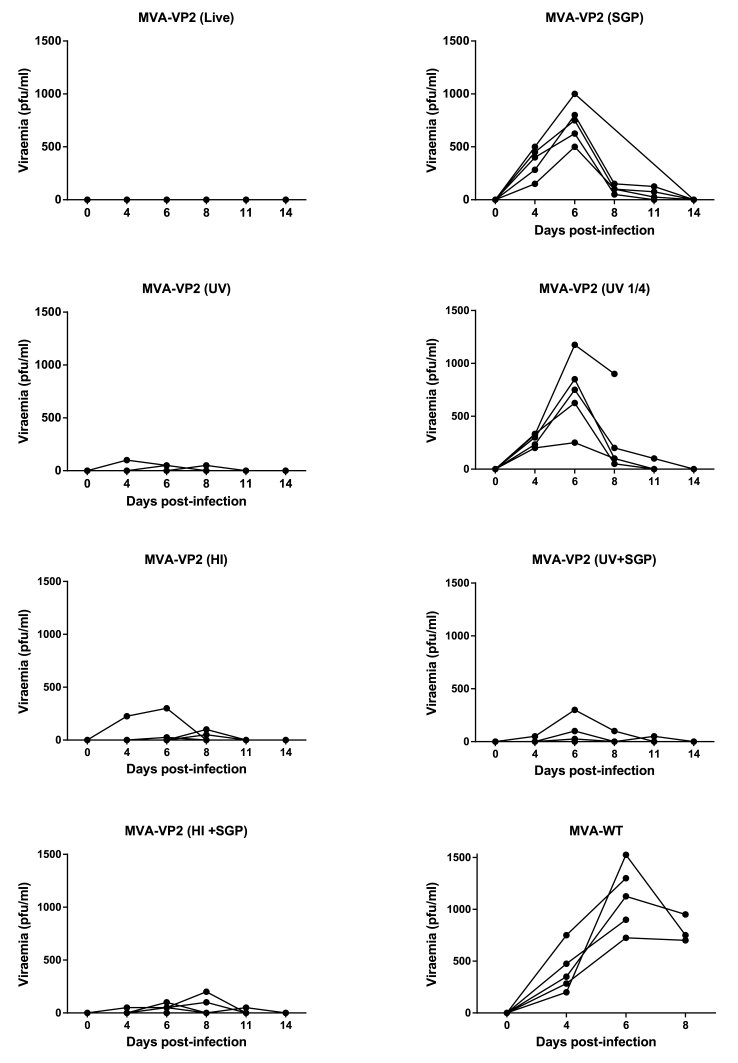
**Viraemia of vaccinated mice following AHSV challenge**. Titres of AHSV-4 in blood of immunized and non-immunized mice were determined at specific time points after challenge. Each point represents the individual virus titres of each animal.

Analysis of viraemia data (Fig. 5) show that AHSV could not be isolated from the blood of MVA-VP2 (live) vaccinated mice and that groups vaccinated with preparations containing inactivated MVA-VP2 (HI or UV) presented very low titre viraemia and only in some individuals. In contrast, the levels of AHSV in blood of MVA-VP2 (SGP) and MVA-VP2 (1/4 UV) vaccinates were more elevated and occurred in all individuals. As expected, blood samples from all negative controls contained AHSV to high titres. Levels of viraemia differed significantly (P < 0.001) amongst mice receiving different vaccines. More precisely, vaccinates could be divided into a low viraemia category [MVA-VP2 (live), MVA-VP2 (UV), MVA-VP2 (HI), MVA-VP2 (SGP) + MVA-VP2 (UV) > MVA-VP2 (SGP) + MVA-VP2 (HI)] and a high viraemia category [MVA-VP2 (1/4 UV), MVA-VP2 (SGP) and MVA (wt)]. Groups within each category do not differ statistically in the levels of viraemia that they presented (P > 0.05).

Collectively the results from the challenge show that groups vaccinated with MVA-VP2 (SGP) and MVA-VP2 (1/4 UV) displayed low levels of clinical protection against AHSV-4, and this correlated with the levels of viraemia displayed by the animals. From the data it can be inferred that the levels of protection observed in the animals also correlated with the presence of pre-formed VP2 in the inoculum of the vaccines. MVA-VP2 (1/4 UV) presented a weak western blot signal corresponding to AHSV-VP2 and this was almost non-existent in the MVA-VP2 (SGP) lane (Fig. 2). When the MVA-VP2 (SGP) vaccine was supplemented with inactivated MVA-VP2 cell lysates, the induction of VNAb and protection against clinical signs, lethality and viraemia were much higher than that induced by the non-supplemented MVA-VP2 (SGP) vaccine. Therefore, it appears that the presence of AHSV-VP2 in the inoculum was a determining factor of immunogenicity of MVA-VP2 vaccines in this study. However, the results also seem to indicate and that the *de novo* synthesis of VP2 in MVA-VP2 infected host cells plays an additional role in immunogenicity since MVA-VP2 (live) vaccines induced the highest protection levels in this study.

### 3.4 Dose-response vaccination experiment with MVA-VP2 (SGP)

The previous experiment indicated that the immunogenicity of MVA-VP2 vaccines relies on the total mass of VP2 protein that is presented to the immune system following inoculation of the vaccine. The VP2 protein in the inoculation site of the vaccinated animals originates from two sources: a) pre-formed in the inoculum; and/or b) expressed from MVA-VP2-infected host cells. In MVA-VP2 (SGP) vaccinated animals, VP2 is almost exclusively derived ‘*de novo’* from MVA-VP2 (SGP)-infected host cells since the inoculum is virtually free from VP2. Since we have observed that an MVA-VP2 (SGP) vaccination dose of 10^7^ pfu/mouse induced a very poor VNAb response, a dose-response experiment was conducted to determine if serum VNAb titres could be induced at all by increasing the dose of the MVA-VP2 (SGP) vaccine.

Thus, 3 groups of mice were vaccinated with MVA-VP2 (SGP) using doses of 10^7^, 10^8^ or 5 × 10^8^ pfu/mouse and the post-vaccination sera subjected to PRNT testing. The mean PRNT titres of vaccinated mouse sera were 1.05, 1.33 and 1.67 for the 10^7^, 10^8^ and 5 × 10^8^ dose groups, respectively. Statistical analysis of the data demonstrated a strong correlation between dose and serum titres (P < 0.001), with every one log increase in dose resulting in a 0.36 (95% confidence interval: 0.28 to 0.44) increase in log_10_ PRNT titre. These data suggest that whilst live MVA-VP2 vaccines containing cell lysates rely on the pre-formed VP2 for inducing a protective VNAb response, *de novo* synthesis of VP2 from host cells plays also a role in MVA-VP2 immunogenicity. The immunoblotting experiment performed with the MVA-VP2 (SGP) vaccines (Fig. 2, right panel) showed that the levels of VP2 present in the inoculum of MVA-VP2 (SGP), even at the highest dose (5 × 10^8^ pfu/mouse), were lower than those of the MVA-VP2 (1/4 UV), which induced a very low VNAb titre in the first vaccination experiment. This indicates that the positive correlation between VNAb titre and the dose of MVA-VP2 (SGP) is probably associated with the increased expression of VP2 in cells of the vaccinated animal. This hypothesis is consistent with results of immunofluorescence experiments performed on MVA-VP2 infected DF-1 cells showing that the numbers of cells expressing VP2 increase proportionally to multiplicity of MVA-VP2 infection (data not shown).

## 4 Discussion

Recombinant MVA viruses have been used experimentally for the prevention of infectious diseases both in humans and in a large variety of animal species (Altenburg et al., 2014, Gilbert, 2013, Moyo et al., 2017, Zhou and Sullivan, 2015). MVA vaccines rely on the expression of relevant immunogenic ‘foreign’ protein genes which are artificially inserted into their genome. The starting point of the immune response following inoculation of an MVA vaccine is the presentation of these immunogenic molecules to the immune system of the vaccinated host. The induction of protective B-cell and T-cell immune responses by means of recombinant MVA virus vaccination has been extensively described in the literature. MVA based vaccines have the ability to induce both arms of the adaptive immune response. Indeed, MVA expressed antigens within ‘professional’ antigen presenting cells of the vaccinated host can be processed via the endogenous and cross-presentation pathways to prime an efficient T cell response. For induction of an antibody response following vaccination it is necessary that B-cell receptors bind to the foreign antigen. However, detailed studies on the mechanisms underlying the induction of antibody responses by recombinant MVA vaccination are not abundant. Some experiments suggest that targeting the foreign antigen to the secretory pathway can lead to improved antibody induction (Yang et al., 1997), whilst others describe strong antibody responses to antigens that are anchored to the cell membrane (Bruder et al., 2010).

We have shown previously that recombinant AHSV vaccines based on expression of AHSV-VP2 (MVA-VP2) are able to consistently induce VNAb responses, cell-mediated immunity and protection against virulent challenge, both in a mouse model and in the equine species (Alberca et al., 2014, Castillo-Olivares et al., 2011, Chiam et al., 2009, Manning et al., 2017). Furthermore, we demonstrated that anti-AHSV protective immunity elicited by MVA-VP2 vaccination is associated mainly, although probably not exclusively, with the induction of VNAb (Calvo-Pinilla et al., 2014, Calvo-Pinilla et al., 2015).

In this paper, we studied the factors that determined the immunogenicity of MVA-VP2 vaccines. Specifically, we studied how the pre-formed VP2 that is present in the MVA-VP2 vaccine inoculum and VP2 that is *de novo* synthesised within cells of the MVA-VP2 vaccinated host influence the induction of VNAb and protection. Our results showed that MVA-VP2 induced immunity relies strongly on the levels of the protein that are delivered to the immune system at the time of vaccination and that both sources of VP2 played a role in the MVA-VP2 vaccine immunogenicity. Thus, all the MVA-VP2 vaccine preparations that contained pre-formed VP2 in their inoculum induced VNAb and protection against disease and viraemia. Protection levels were reduced when the levels of VP2 were reduced or absent in the inoculum of live MVA-VP2 vaccines (at a dose of 10^7^ pfu/mouse). However, the protective capacity was restored when the inoculum of such vaccines was supplemented with pre-formed VP2, as was observed in the case of mice vaccinated with MVA-VP2 (SGP) + MVA-VP2 (UV) or MVA-VP2 (SGP) + MVA-VP2 (HI).

However, sterile immunity was only induced by MVA-VP2 (live) vaccines, which contain pre-formed VP2 and also express VP2 in host cells following vaccination. The maximum level of protection in MVA-VP2 (live) vaccinates could be explained by the highest VNAb titres reached by these animals at the time of challenge. We determined previously (Calvo-Pinilla et al., 2014, Calvo-Pinilla et al., 2015) that protection against AHSV in MVA-VP2 vaccinated mice correlated strongly with VNAb titres at the time of challenge. It is also possible that in the present study the induction of cell-mediated immune responses by MVA-VP2 vaccination contributed to the highest level of protective immunity achieved by MVA-VP2 (live), since previous experiments showed that when administered as a live vaccine, MVA-VP2 inoculation induces partially protective cell mediated immune responses (Calvo-Pinilla et al., 2014). Additionally, the expression of VP2 within host cells is likely to best preserve the native epitope structure of the protein and its conformational VNAb epitopes. This is unlikely to occur in the pre-formed VP2 of the MVA-VP2's inoculum. Indeed, the VP2 protein in the inoculum of MVA-VP2 (HI) and MVA-VP2 (UV) vaccines was subjected to physical treatments that were likely to have affected VP2's tertiary structure.

Any of the mechanisms alluded to above could only occur if expression of VP2 within cells of the MVA-VP2 vaccinated host actually took place. In the first experiment described in this paper, a dose of 10^7^ pfu of MVA-VP2 (SGP) induced very low levels of VNAb (just below a titre of 10) and it was not clear whether the MVA-VP2 immunogenicity depended exclusively on pre-formed VP2 present in the inoculum. However, in the dose-response experiment we showed that increasing doses of MVA-VP2 (SGP) correlated with increasing VNAb titres in the vaccinates sera. This strongly suggests that VP2 is expressed from within host cells and is effectively processed and presented to the immune system to induce VNAb and possibly cell-mediated immunity. Therefore, it can be concluded that both pre-formed VP2 in the inoculum and *de novo* expression of VP2 from MVA-VP2 infected cells of the vaccinated host contributed to the induction of the highly protective immune response of MVA-VP2 vaccination, but that if administered in a purified form, MVA-VP2 would need to be administered at higher doses than when it is administered as a crude lysate.

With regards to the reduced immunogenicity observed in mice vaccinated with MVA-VP2 (SGP) vaccine, it is worth noting that other poxvirus vector vaccines are formulated with an adjuvant (Draper et al., 2008). Thus, Carbopol, an efficacious adjuvant for the equine species, is used to formulate Canarypox-based vaccines for equine influenza, herpesvirus and West Nile virus (Draper and Heeney, 2010, El Garch et al., 2008, Minke et al., 2006, Minke et al., 2007). Notably, a prototype vaccine against AHS based on Canarypox was also formulated with this adjuvant (Guthrie et al., 2009). The exact mechanisms by which Carbopol exerts its adjuvant effect on this type of vaccine are not known, but it is possible that these effects can also be replicated in the case of MVA-VP2 vaccines.

The observations made in our study open new avenues for improving the efficacy of MVA based vaccines for infectious diseases in general and for AHSV in particular. Consequently, it would be interesting to test whether improved antibody responses could be achieved by enhancing the secretion of the antigens from MVA-VP2 infected cells, which would facilitate B-cell receptor binding of the antigen. Targeting the MVA-expressed antigens to the extracellular space might also contribute to preserving the native structure of relevant conformational epitopes. In addition, it would also be equally interesting to examine the importance of the antigen processing and MHC presentation pathways within host cells infected with MVA vaccines to direct more effectively the induction of T-cell responses. In previous experiments we showed that the main effector mechanism of immunity of MVA-VP2 vaccines was mediated by VNAb, though other studies (de la Poza et al., 2013, El Garch et al., 2012) and the results presented in this paper suggest cellular immunity could have an important complementary protective role.

The use of recombinant MVA viruses expressing AHSV-VP2 have been shown to be a suitable approach for developing safe, efficacious AHS vaccines with DIVA (Differentiation of Infected from Vaccinated Animals) capability (Chiam et al., 2009, Castillo-Olivares et al., 2011, Alberca et al., 2014, Calvo-Pinilla et al., 2015, Calvo-Pinilla et al., 2015). Furthermore, recent studies suggest MVA-VP2 could be used to develop polyvalent AHS vaccines (Manning et al., 2017), an essential feature if the vaccine is to be used in an endemic country. MVA-VP2 vaccines could be manufactured commercially and this largely depends on the final design and formulation of the vaccine which will be determined by some of the conditions and parameters examined in this study.
